# A Qualitative Phenomenological Philosophy Analysis of Affectivity and Temporality in Experiences of COVID-19 and Remaining Symptoms after COVID-19 in Sweden

**DOI:** 10.1007/s10912-024-09858-w

**Published:** 2024-06-26

**Authors:** Kristin Zeiler, Sofia Morberg Jämterud, Anna Bredström, Anestis Divanoglou, Richard Levi

**Affiliations:** 1 Department of Thematic Studies: Technology and Social Change, and the Centre for Medical Humanities and Bioethics, Linköping University, Linköping, Sweden; 2 Institute for Research On Migration, Ethnicity and Society, and the Centre for Medical Humanities and Bioethics, Linköping University, Linköping, Sweden; 3 Department of Rehabilitation Medicine and Department of Health, Medicine and Caring Sciences, Linköping University, Linköping, Sweden

**Keywords:** long COVID, patient experiences, existential feelings, fear, rehabilitation

## Abstract

This article explores affectivity, temporality, and their interrelation in patients who contracted COVID-19 during the first wave of the pandemic in Sweden and with symptoms indicative of post-COVID-19 Condition (PCC) that remained one year after the infection. It offers a qualitative phenomenological philosophy analysis, showing how being ill with acute COVID-19 and with symptoms indicative of PCC can entail a radically altered self-world relation. We identify two examples of pre-intentional (existential) feelings: that of listlessness and that of not being able to sense what is real and not real, both of which, in different ways, imply a changed self-world relation. We offer an analysis of intentional feelings: how the fear of not “returning” to one’s previous self and the hope of such a return weave together the present and the absent, as well as the past and the future, in ways that make the future appear as constricted, disquieting, or lost. We argue that a phenomenological differentiation among experiences of living with symptoms indicative of PCC—through attention to the way intentional affectivity and pre-intentional affectivity help shape the embodied self’s attunement to the world—is apt to yield a better understanding of the variations within these experiences and contribute to clinical practice.

## Introduction

By September 2022, the COVID-19 pandemic had resulted in over 6.5 million deaths, according to the World Health Organization (WHO 2022). The disease continues to have unforeseen effects on individuals and groups of people, not the least among those with Post COVID-19 Condition (PCC), sometimes also referred to as long COVID. PCC is defined as “the continuation or development of new symptoms three months after the initial SARS-CoV-2 infection, with these symptoms lasting for at least two months with no other explanation” (WHO 2022). This article offers a qualitative phenomenological philosophy analysis of affectivity, temporality, and their interrelation in patients’ experiences of falling ill and being hospitalized with COVID-19 during the first pandemic wave and then experiencing persisting symptoms for more than two months. At the time of the interviews, PCC was not yet established as a formal diagnosis in Sweden. However, it can retrospectively be established that all interviewees fulfilled the diagnostic criteria for this condition.

The article’s aim is twofold: (1) to understand patients’ lived experiences of falling ill with and living with remaining symptoms of COVID-19 at a time of thorough societal and medical uncertainty as regards what COVID-19 could entail, short- and long-term, and (2) to demonstrate the value of attending to both intentional and pre-intentional affectivity in clinical care.

By so doing, the article contributes insights into medical humanities and the qualitative phenomenological philosophy of illness experiences. From early on in the pandemic, medical humanities studies emphasized the role of patient knowledge in the conceptualization and understanding of COVID-19 (e.g., Callard and Perego 2021) and pointed to “layered temporalities” of COVID-19 narrations focused, for example, on fragmentation and chronicity (Howell 2022). Further, past studies on the lived experience of symptoms following COVID-19 or of PCC (or Long-COVID) have included experiences of a wide range of symptoms, how the illness fluctuated over time, a sense of shame related to having remaining symptoms, fear (e.g., of not recovering and losing one’s job), and how living with the remaining symptoms impacted on one’s self-understanding (Cooper et al. 2023; Callan et al. 2022; Macpherson et al. 2022), and, for example, feelings of guilt (Engwall et al. 2022). Other studies have brought out patients’ sense of “becoming anonymous” while being admitted to an intensive care unit (ICU) during the pandemic, when extreme isolation protocols were in place (Køster 2023), and different forms of objectification (including self-objectification) in the ICU during the pandemic (see the article by Køster, Fernandez, and Andersen in this Special Issue). This article contributes to past medical humanities research on COVID-19 by exemplifying and calling for attention to be given to illness experiences that defy a neat temporal closure and by engaging with the phenomenological understanding of affectivity as central to subjectivity and as shaping and being part of how the self experiences itself, others, and the world as a field of possibilities.

Initially, we provide a brief context of the pandemic as it played out in Sweden and describe phenomenological distinctions pertinent to the study. The analysis proper is presented in three parts. First, we attend to interviewees’ experiences of falling ill with COVID-19 (at a time characterized by a lack of diagnostic capacity and uncertainty as regards the symptomatology and management of the disease) and the juxtaposition of chronological time within a temporal horizon of experienced narrowed possibilities. Thereafter, we make use of the distinction between intentional affectivity and pre-intentional “existential feelings” (Ratcliffe 2011). Intentional affectivity refers to feelings/affects that have an “aboutness,” meaning that they are intentionally directed to (and about) something, be it a situation, person, thing, or something else. “Existential feelings” is Matthew Ratcliffe’s (2011) term. Such feelings are not about something specific and can instead be described as affective “background orientations” that structure one’s experience as a whole (Ratcliffe 2011, 2). In this sense, they are pre-intentional and “disclose the world as a whole” and both open and constrain the very “range of the intentional feelings that can manifest” (Fernandez 2014, 598). We identify two examples that we see as qualifying as candidates for the category of existential feelings: that of listlessness as a thorough yet subtle form of apathy and that of (temporarily) not sensing what is real and not real. We also show how intentional feelings of fear of not being able to “return” to one’s pre-COVID self while experiencing persisting symptoms and of hope of such a return help shape how the past, the present, and the future are experienced. Further, based on the analysis, we suggest that a phenomenological differentiation among experiences of living with remaining symptoms after COVID-19, through attention to the way intentional and pre-intentional affectivity (specifically existential feelings) may shape the embodied self’s attunement to the world, is apt in order to better understand some of the variations within these experiences, and thus contribute to clinical practice. Particularly, pre-intentional existential feelings can imply a radically changed self-world relation and yet may easily be disregarded in clinical care. However, we acknowledge that, for example, the feeling of “unrealness” identified in this study resonates with features of, for example, some dreamlike experiences previously reported by ICU-treated patients (as analyzed by, for example, Køster 2023). Further studies are needed before it is possible to take a stand on the question of whether experiences such as those identified and analyzed in this article are common for COVID-19. This notwithstanding, the narrated experiences analyzed in this article are important to acknowledge for a better understanding of the possible ways of experiencing the condition and its aftermath.

## The Swedish Context

The first pandemic wave in Sweden occurred in the spring of 2020. At this time, the Swedish health care situation (as in most other countries) was characterized by a lack of resources regarding diagnostic capacity, insufficient availability of protective gear, insufficient number of ICU beds, and uncertainty as regards optimal medical management. In addition, there was insufficient coordination between national, regional, and municipal levels of government (SOU 2021, 89). The Public Health Agency of Sweden (Folkhälsomyndigheten) recommended COVID-19 tests only for patients admitted to hospital, for suspected cases in persons living in institutional care settings (primarily elderly care), and for suspected cases among staff in health care and social care (Folkhälsomyndigheten 2020a, 10; 2020b). Others with suspected COVID-19 were advised to stay at home and were not tested for the disease.

As for the presence of lingering symptoms post-discharge from hospitalization due to COVID-19 during the first pandemic wave, at least 70% of such patients in one of 21 Swedish health care regions (Region Östergötland, RÖ, population approx. 500,000) reported such symptoms at four months, with 40% having problems affecting daily life to some, high or very high degree, and thus indicative of rehabilitation needs (Divanoglou et al. 2021). Apart from symptoms attributable to the respiratory tract, patients experienced a wide array of other symptoms (e.g., fatigue, pain, cognitive impairment, and anxiety) (Divanoglou et al. 2021; Wahlgren et al. 2022).

At the time of the interviews in this study (April–October 2021), the Swedish context was influenced by three events. First, the Swedish government established a new temporary pandemic law that granted the government the authority to implement binding infection control measures during the pandemic. The law, which came into effect in January 2021 and was valid during the time of the data collection, aimed to reduce the spread of the virus and included restrictions to mitigate crowding and transmission (SFS 2021, 8). However, unlike some other European countries, it did not entail a complete travel ban or total lockdown. Second, concurrent with the pandemic law, the Public Health Agency of Sweden began recommending vaccination against COVID-19, and during the spring of 2021, vaccines started to be offered to prioritized groups, such as the elderly (Folkhälsomyndigheten 2023). Third, in 2021, the situation within the Swedish health care system also became increasingly challenging as health care providers encountered a growing number of patients experiencing post-COVID symptoms, and this situation created a need for guidelines, which the National Board of Health and Welfare (Socialstyrelsen) released in the spring of 2021. In these guidelines, the Board acknowledged that there was not yet any evidence-based approach for investigating, treating, and rehabilitating individuals with symptoms after COVID-19, and they also concluded that the patient group was heterogeneous and that the symptoms varied widely (Socialstyrelsen 2021b). Notably, the lack of widespread COVID-19 testing in Sweden made it challenging to definitively diagnose PCC in some cases. However, the guidelines state that PCC could also be diagnosed in the absence of confirmatory laboratory testing based on the clinical picture (Socialstyrelsen 2021b). By June 2021, 11,000 individuals in primary care in Sweden had been diagnosed with the condition (Socialstyrelsen 2021a).

Throughout the article, we use the formulations “remaining symptoms after COVID-19 infection” and “symptoms indicative of PCC” as synonyms.

## Methods and Research Ethics

This study comprises interviews performed with 13 patients previously hospitalized due to COVID-19 during the first pandemic wave in RÖ. Interviewees were recruited from the subgroup of patients who had persistent symptoms after COVID-19, with a significant negative impact on daily activities at a five-month post-discharge assessment (Wahlgren et al. 2022).^1^ Specifically, inclusion criteria demanded laboratory-verified SARS-CoV-19 infection and debut of symptoms in conjunction with the infective episode. Exclusion criteria demanded that the informant did not suffer from any comorbid conditions to which symptoms could be attributed. This included, for example, the exclusion of informants with clinically significant depression prior to the COVID-related hospitalization. Variation among interviewees was sought as regards sex, age, ethnicity, educational level, and disease severity.^2^ The interviews were semi-structured, open-ended, and qualitative in character, focusing on interviewees’ experiences of falling ill, being hospitalized, and being in a recovery phase after COVID-19 infection. More specifically, the interview guide contained the following sections: (1) falling ill and being ill with COVID-19 (what happened/how did it feel to fall ill; how did they come to know that they had COVID-19; how did they experience this; how did they come to be hospitalized with COVID-19, what happened and how did they experience this; how did they experience the health care they received); (2) rehabilitation after COVID-19 (how was it to return home after the hospitalization; did they need rehabilitation; how have they experienced rehabilitation, if any, after COVID-19); (3) the impact of having fallen and being ill with COVID-19 on their everyday life and challenges when being ill with COVID-19 in the encounter with health care. Each interview was recorded, transcribed verbatim, and pseudonymized. Two interviews were performed through a professional interpreter. Three patients chose to be interviewed together with a close other, while 10 chose individual interviews. Interviews took place 12–15 months post-hospital discharge.

Subsequent analysis took place in five steps (compare the five steps of qualitative critical phenomenology in De Boer and Zeiler [2023]). The first step comprised a thematic analysis (Braun and Clarke 2006), which entailed line-by-line coding of the material and the identification of themes and sub-themes. One of the identified themes was that of temporality and the interrelation between affectivity and temporality. This was not a predefined theme to be asked about in the interviews but a theme that was identified through the first analytic step. The second step entailed engaging with phenomenological philosophy in the light of the identified theme—in this case, with phenomenological literature on illness experiences and on affectivity and temporality. The third step comprised re-reading and re-coding the interviewees’ narrations and accounts via the phenomenological literature and re-grouping codes and sub-themes into more phenomenologically centered themes, which might be called phenomenologically oriented themes. Steps two and three comprised a dialogical process where data and literature were read in relation to each other, as asking questions to each other. The fourth step entailed identifying quotes within the phenomenologically oriented theme that may be discussed or interpreted more elaborately. The fifth, final step entailed contributing to conceptual discussions in phenomenology, including discussing different interpretations of the empirical material and whether some of these required elaborations of existing concepts or the coining of new concepts to grasp what was at stake in the empirical material. Through these steps, the article exemplifies how qualitative and phenomenological philosophy inquiry can be combined (for discussion and examples of different ways of doing qualitative phenomenological philosophy, see, e.g., De Boer and Zeiler [2023]; Køster and Fernandez [2023]; Køster [2020]; Zeiler and De Boer [2020]).

SJM and AB performed the interviews; KZ, SJM, and AB conducted the first step of the analysis; KZ took the lead in the second to fifth steps together with SJM. KZ took primary responsibility for writing the article. AD and RL contributed medical and health care expertise, AD oversaw the recruitment of the interviewees, and RL took primary responsibility for the section “Clinical Relevance.” All co-authors contributed to writing the article. Research ethics approval was obtained from the Swedish Ethical Review Authority (Dnr 2020–05662). All interviewees—patients and close others—gave their written informed consent to be part of the study.

## Phenomenology: Embodiment, Temporality, and Affectivity

Phenomenological philosophy starts from an understanding of the self or subjectivity as embodied and embedded in a world and in relations with others. It attends to structures that make possible and help shape experience and to how, for example, the world, things, oneself, embodiment, or time appear to the self in experience. Following the work of Maurice Merleau-Ponty ([1945] 2013), for example, the embodied and embedded self is understood as being directed towards and perceiving the world from a specific position and perspective at the same time as being part of and engaging with the world. The world is not the “objective” world of the natural sciences with its focus on cause and quantifiable measurements but one of meaning. Making use of the distinction between, on the one hand, the body as subjectively lived and as an embodied first-person perspective on and experience of the world and oneself (*Leib*) and, on the other, the body as an object (*Körper*), phenomenology underlines that these perspectives help to form each other and that one’s experience of one’s own lived body also involves the experience of one’s body in its object-like dimensions and as seen by others. Phenomenological studies have also attended to how the body in everyday experiences can recede from the self’s attention, and how a pre-reflective awareness and a tacit system of sensorimotor capabilities enable smooth and seamless coordination of bodily movements in space and time, as well as in relation to others, and help open up the world as a field of possibilities for the self (Merleau-Ponty 2013). This is what makes the smooth alignment of one’s body with its environment possible. The tacitness and seamlessness can also change in experiences of pain and illness where the “thing-like” side of embodiment takes precedence in self-awareness, the hurting body or body part standing forth as an “it” that one cannot but attend to and the world appearing unfamiliar, dangerous, or inaccessible (e.g., Leder 1990; Carel 2008; Zeiler 2010).

Detailed accounts have been given of the intertwinement between embodiment and temporality, such as in analyses of how past repeated ways of acting and interacting can sediment into our habitual bodily mode of being and help shape the self’s perceived field of possibilities in the present (Merleau-Ponty 2013). The intertwinement between embodiment and temporality has also been discussed in relation to the distinction between “implicit” and “explicit” temporality (Fuchs 2005). Implicit temporality refers to the experience of time when we are absorbed in what we are doing. When this is the case, we may neither attend to embodiment (for example, to how to move in space) nor to the experience of time (which is not to say that we are not at all aware of our bodies or time when being immersed in this way).^3^ In this mode of being, we “are ‘inside time’” (Wehrle 2020, 506). Explicit temporality refers to the experience of time when it becomes a thematic object of attention, as when something happens that disrupts our previous absorption in what we were doing, and we note how time has flown by. Quantifiable time, by contrast, refers to time as measured in seconds, minutes, hours, etc. from a third-person perspective.

As well as temporality, affectivity can be understood in terms of “modes of bodily attunement to, and engagement with, the lived world” (Fuchs 2013, 2). Affectivity, as an umbrella term for feelings, emotions, and moods, is that through which the world and things in it appear to us in distinct ways and matter to us; different affective modes shape how we experience the world or things in it, while also shedding light on the constitution of the self as a being that can be affectively attuned to the world. Further, we make use of the distinction between intentional affectivity, for feelings that are directed to something specific (e.g., fear of specific things or situations that can shape distinct self-world relations [see, e.g., Ahmed 2004]), and pre-intentional affectivity that lack intentional reference (is not about something specific, such as anxiety that forms one’s perception and experience of oneself and the world, as a whole).^4^

Engaging with past phenomenological work on affectivity, Matthew Ratcliffe has coined the term “existential feelings” for pre-intentional affectivity characterized by a distinct relation between the self and the world: existential feelings are “ways of finding oneself in a world” (Ratcliffe 2011, 2). For Ratcliffe, existential feelings are not directed at specific things or situations but are “background orientations” and “bodily states of which we have at least some awareness” (Ratcliffe 2005, 2). Existential feelings can “determinate what kinds of intentional states it is possible to have” (Ratcliffe 2010, 604), imply an “altered sense of relatedness” between self and world that affects the manner in which they both are experienced (Ratcliffe 2009, 180), and help shape and constrain *all* experiences. What is felt in existential feelings is “a changed relationship to the world as a whole, an alteration in the possibility space that one finds oneself in” (Ratcliffe 2011, 124; see also Ratcliffe 2005, 45). Some examples of existential feelings are feelings of familiarity and unfamiliarity, where the point is not that there is a lack or absence of the feeling of familiarity but a distinct “feeling of unfamiliarity” (Ratcliffe 2011, 148) and feelings of unreality and reality.^5^

Further, Anthony Fernandez suggests that the analysis of existential feelings can be further specified via the use of the distinction in Heidegger between structural and modal changes, where changes in the structure of human existence include a change in one’s very capacity or the degree to which one is able to be affected, at all. Modal changes, on the other hand, include profound changes in one’s way of being—*how* one is—affected and finding oneself in the world—for example, changes in the “particular mode of attunement in which we find ourselves” (Fernandez 2014, 598).^6^

## Layered Temporality and Uncertainty when Falling Ill with COVID-19

For many of the interviewees, falling ill with COVID-19 started with mild symptoms such as headache, fever, dry cough, or asthma-like symptoms. For all of them, the condition gradually aggravated during a period of one to two weeks prior to hospitalization. Most of them tell of how they repeatedly called their local primary care center or the national telephone line for health care advice, describing their symptoms, noting how many days they had had a particular symptom, and asking for advice or for an appointment for assessment. They describe how they were told to remain at home and call back if their situations further deteriorated. A few examples are indicative: interviewee Anders explains that he had a high fever and severe cough and that he called his primary care center and was told that he is young, that there is nothing to worry about, and that “you can’t come here anyway … try to stay still, though you can move around indoors” and “take something against the fever.” As the days went by, he describes an emerging feeling of “panic, of not knowing what is happening in one’s body … I have no energy to go to the toilet … no energy to walk up the stairs.” When once more calling the primary care center, and this time being asked if he has respiratory difficulties, he describes how he “broke down and said, ‘I don’t know what respiratory difficulties *are.*’” Anna also describes having had a fever and severe coughing and how she became breathless when walking short distances. She describes her illness experience as “pure hell.” She also explains that she knows how an asthma attack feels as she has had asthma for 20 years. This time, however, she says she “can’t interpret the signals” from her body. When calling health care, she is told to wait at home, she says, until she eventually gets very angry and tells the nurse at the health care advice line that it feels like “an elephant is sitting on my chest” and that this is not an asthma attack. Muhammed, as one further example, describes how COVID-19 “came as a wave and we all got sick.” He narrates how he had stayed at home for 10 days, with symptoms, when he called the primary care center, who responded: “Stay at home; it’s nothing serious.” “They didn’t want any patients,” Muhammed reflects, adding, “but then it got very serious.”

As exemplified above, interviewees narrate an uncertainty regarding what would qualify as severe enough symptoms to be admitted to the hospital. Further, their experiences of being ill are interwoven within the socio-cultural context in which they took place. The first pandemic wave in Sweden was a time of uncertainty, not only in terms of the globally uncertain knowledge about the SARS-Cov-2 virus but also a more specific uncertainty among the general population in Sweden of not being able to test for COVID-19 before being deemed in need of hospital care (Folkhälsomyndigheten 2020b). Interviewees’ narrations of their symptoms—their frustration (as in “breaking down,” above) when not knowing whether or how their experiences should be translated into medical terminology (“what respiratory difficulties are”), and the way they note how long they have been home with aggravating symptoms—need to be contextualized against this background.

Further, interviewees worry about what is happening in their bodies, and they note how even simple tasks require careful planning and an energy that they no longer have. In such narrations, the experienced field of possibilities can be understood as narrowing in two senses: possibilities can be brought to one’s attention as impossible or as most difficult (i.e., as standing out in terms of what one can no longer do, as in the excerpt on no longer being able to walk up the stairs) *or* they may no longer appear to the self, at all. The latter resonates with yet another interviewee’s—Gabriel’s—account of how, as symptoms aggravated, pain was felt “everywhere, in the organs and the body, in the head, in the thoughts,” thoroughly altering the perceived field of possibilities, narrowing the focus to the body in pain.

This brief analysis allows for two points. First, the way interviewees count the days while noting what they can no longer do can be understood as exemplifying “layered” temporalities of the pandemic in the form of quantifiable time (as in counting the number of days with a certain symptom) being juxtaposed into a temporal horizon of narrowing possibilities. This is something we will return to in relation to experiences of fear. Second, the lived body as a body-world relation can be disrupted not only due to severe illness but also if one’s world is radically altered, as during the pandemic, with restrictions and other measures put in place to prevent the spread of infection (e.g., when these disrupt previously habitual ways of engaging with others and help shape how things appear as possible or impossible). For the interviewees who experience gradually worsening symptoms, the gradual disruption of their lived bodies can be understood as double-layered: they experience growing severity of illness in a radically altered world due to a disease soaked with uncertainty. In addition, the way Sweden was portrayed and debated as having opted for a distinctly different position vis-à-vis recommendations for how to act, on an individual as well as societal level, can also feed into the felt uncertainty. Not only was there global uncertainty as to what COVID-19 entailed, but Sweden was also often described as having chosen an unusual path.

## Pre-intentional Existential Feelings and Temporality After Hospitalization

For Ratcliffe, “existential feelings” is a distinct phenomenological category that constitutes “a sense of relatedness between self and world,” and it is this “self-world relation that is described as “ ‘feeling’ a certain way” (Ratcliffe 2009, 180–81). In existential feelings, the *sense* of one’s anchoring in the world is at stake: what is “felt is a changed relationship to the world as a whole,” and the changed sense of the relation between self and world shape how both are experienced (Ratcliffe 2009, 124). A feeling of reality as slipping away, of reality being attenuated or objects being “stripped of the usual familiarity that surrounds them” and then “appearing as alien and without meaning,” or a feeling of “a lack of connectedness, an absence of the usual familiarity, of significance, of belonging,” or of “emptiness” are but some examples (Ratcliffe 2011, 63 and 65). Further, existential feelings, in Ratcliffe’s analysis, are bound up with one’s activities in the sense that they “constitute the general *space* of possibilities that shapes ongoing experience and activity” (122, italics in the original) and have a temporal structure in the sense of “a changed sense of possible activities that one *might* pursue” (125, italics in the original). In our study, interviewees’ experiences of listlessness (the Swedish *håglöshet*) and of not sensing what was real and non-real can, we suggest, be understood as existential feelings.

After being discharged from the hospital, all interviewees describe themselves as focused on how to recover. This was typically formulated in terms of a longing for a “return” to one’s previous self. For many of the interviewees, such a return did not come as easy as envisioned and hoped for. Instead, as some of them narrate, they experienced a growing tiredness and lack of energy that had specific qualities of them not feeling that things mattered to them and not being able to make themselves care. This feeling was also described as prevailing over time.

These interviewees describe how they experience themselves as no longer having the energy to take initiative. This is the case, for example, when interviewees Marianne, Per, and Bengt, in different ways, describe how they felt that “nothing mattered.” As a few examples, Marianne tells of how she sees papers lying around but cannot bring herself to remove or sort them, and Per describes how he “postpones things he must do,” even if they are “easy.” He is tired, he has lost the “ability to take initiative,” and he experiences himself as “listless.” Per and Bengt describe a felt “resistance,” an experience of not having the energy to engage in activities, and a sense of “*oföretagsamhet*,” a lack of initiative. The Swedish formulation *oföretagsamhet* can have connotations of oneself not caring enough to act; one cannot bring oneself to act, and one does not take action despite seeing that action could be good (as in sorting the papers). We use the term *listlessness* to capture this pre-intentional affective phenomenon: when narrated in the interviews, listlessness is not about specific things or activities (even though specific examples sometimes are given) but rather seems to imply that one’s self-world relation appears as drained of energy and vibrance. It is not primarily that the perceived space of possibilities is experienced as narrowed (i.e., as would be the case if there are fewer things people experience that they can do) but that the *sense* of that space has changed, including its sense of temporal openness as a space of possibility that one might be drawn into.

Phenomenologically, scholars have discussed how things can lose the “allure” (e.g., Husserl 2001, 196) that previously drew in someone’s attention and no longer “solicit us to act in ways that actualize enticing [perceptual and practical] possibilities” (Ratcliffe 2012, 119). The point here is not that projects appear as not at all practically significant in the sense of being experienced as “relevant in the context of a set of goals, projects and values” (Ratcliffe 2012, 119 and 124), but there is no experienced drive, and things are not experienced as mattering. To relate to the example of the interviewee, there is a phenomenological difference between the paper appearing as something I would sort even if it is simply part of everyday routines, on the one hand, and the experience of not being able to make oneself care, on the other. Things one previously just did now instead appear as what one cannot make oneself care about; the feeling of significance, the mattering, no longer opens up and shapes one’s relationship to the world. For the interviewees, this makes them note how thoroughly their relation to their environment has changed.

Further, experiences of listlessness can be phenomenologically differentiated from experiences of having “been on the wrong track” for so long, after being discharged from the hospital, that one now feels that “this will never work.” The latter, however, is how the interviewee Ida describes her experience. Ida tells of how she feels that she has lost her energy and direction. “It feels quite dark, actually,” she says, adding that she has lost her “joy of living” and the “sparkle in her eyes.” She adds that the sparkle in her eyes is no longer there because all that she can no longer do has taken her aback so thoroughly—she can no longer walk, no longer go to the toilet, no longer put her pants on by herself. “I cry more after Corona; I cry more easily. And I don’t know why I cry.”

Ida’s narration exemplifies an experience of frustration or despair in the very experience of still being committed to—wanting and trying to engage in—various activities. In such a case, if things are no longer experienced as relevant in the context of one’s goals and projects, this is because the goals and projects no longer appear possible at all, given one’s specific bodily situation. Goals and projects no longer appear attainable, and this very felt unattainability is painfully weighty: goals and projects have not receded but instead appear precisely as not within reach. In such a case, one’s body has changed so thoroughly that many or even most activities are experienced as outside one’s field of perceived possibilities.

We also identify another possible example of an existential feeling in the interviewees’ narrations: a difficulty in sensing what is real and not real. This was a particular feature of narrations by interviewees who had been on mechanical ventilation during hospitalization. For the interviewees with this experience, the difficulty in sensing what is real and not real first occurred while at the ICU, when being told that what they had experienced when on the ventilator was not real (as they had previously assumed that it was). They described how they gradually came to accept this. However, the interview material also contains a recurring experience of not sensing what is real and not real when having been discharged and living with remaining symptoms indicative of PCC in one’s home.

In our study, the interviewees Anders and Anna describe how they remember vivid detail and how they experienced themselves as present—as noticing and seeing and actively taking part in activities—while being on the ventilator. In this way, Anna describes how she experienced her son coming to visit her at the hospital and telling her that her partner had died. This devastated her. After being weaned off the ventilator, she recalls, she became angry with the health care staff, who brought her mobile phone to her and said that her partner was not dead and played voice messages to her from her partner. She found this a very cruel way to trick her into believing that he was not dead by playing pre-recorded messages from before his death. Eventually, when she was strong enough to talk to him over the phone, realizing that he was alive, she describes how she “cried and cried.” Her partner is present during the interview, and he adds that when he was allowed to visit, Anna could still not talk but only to wheeze out that she wanted him to come close and hug her while also wheezing, “You’re not alive.” He recalls that he responded, “Oh, yes, I am, I am here now,” and to which Anna replied, “Yes, you are.” “It was,” he adds, “as if she had to make that thought enter her head.” Anders also narrates his experiences while on the ventilator as vivid and very real. He explains that he took part in great parties in the hospital ward and got to know all the staff very well. Further, after being weaned off the ventilator, he told some of the nurses that if he were ever to change jobs, he would become an ICU nurse working night shifts because they had had such a great time together—the best of parties. He describes his shock when being told by one of the nurses that what he had experienced “actually was not real”—that is, it never happened.

These interviewees’ vivid experiences while being on the ventilator, their difficulty in discerning between what was real and not real when being at the ICU, and their gradual acceptance of what they had experienced while on the ventilator as not real resonates with findings in other studies, where critically ill patients, admitted to ICUs, describe themselves as believing what they experienced as real and themselves as awake and present at the time (Engström et al. 2022) and feeling distressed (Boehm et al. 2021). This is also the case in Allan Køster’s (2023) analysis of “becoming anonymous” in ICU wards during the pandemic, which brings out how the pandemic experience at ICUs implied open-ended admissions combined with lack of prognosis, far-reaching isolation, and, for the personnel, the use of extensive personal protective equipment. For some of the patients in that study, the ICU experience included the experience of a dream-like state and difficulty or even “breakdown” in the ability to distinguish between what was a dream and what was not (Køster 2023, 1044). Such research contains descriptions of experiences that at least partly fit how derealization is defined in the current *Diagnostic and Statistical Manual of Mental Disorders* as “experiences of unreality or detachment with respect to surroundings (e.g., individuals or objects are experienced as unreal, dreamlike, foggy, life­less, or visually distorted)” (American Psychiatric Association 2013, 1044).^7^

Further, the interviews in this study also contain narrations of experiences that were not characterized by reality being attenuated or appearing as alien or stripped of meaning, and that took place when living with remaining symptoms after COVID-19 in one’s home. Interviewee Anders tells of such experiences. As Anders’s interview unfolds, he describes having difficulty sensing or discerning between what was “really true” and what was not. This did not only occur at the ICU but also when waking up at home during the night. Anders recalls how this happened a long time after being discharged from the hospital and over a longer period of time. He tells of how he and his partner could fall asleep in the evening, and he could wake up sometime later in the night, turn on the light, and not know what was “real” and “really true” and what was “not real.” When having these experiences, he says, he would ask: “Where am I? Am I at home? Is it certain that I am at home, or am I dreaming this too?” and “Am I *really* experiencing this?” He describes this as thoroughly disconcerting and as part of experiences of panic.

We suggest that feelings of unreality in terms of attenuation, alienation, or objects losing their familiarity and meaning do not resonate, at least not fully, with this account. A few things are noteworthy. First, this narration is not about a distorted experience, and while it may be dream-like, the interviewee does not narrate specific dream-like features, but the narration instead centers on the difficulty of sensing what is real or not—what is a dream. This narrated difficulty, we suggest, may be interpreted in terms of a temporally localized difficulty or breakdown of the ability to sense what is real or not real. Second, the affective experience of not sensing whether something is real or not real (occasionally also formulated as “not true”), above, is framed in this way (real/not real) and not in terms of *un*reality. This linguistic difference may point to a phenomenologically relevant difference between the feeling of unreality in terms of reality as slipping away or of objects having an alien character, on the one hand, and that of feelings where reality is not experienced in this way and yet the very sense of the real and the not real is at stake.^8^

Further, the specific interviewees’ narration, above, of waking up and wondering: “Am I really experiencing this?” is not expressed as directed towards specific objects or things that appear as unfamiliar. This could, of course, be the case, though not expressed, which leaves two possible interpretations: the narrated experience might exemplify an affective experience that is about uncertainty in discerning *specific things* as “real” or “not real” even though that is not narrated or an affective experience where the very *sense* of the not real and the real is at stake. However, what seems central, in this case, is the way the experience *at* the hospital of learning that what was experienced as real was not real *resonates* in this person’s experienced field of possibilities while living with remaining symptoms at home (as in asking “Am I really at home or am I dreaming this *too*?”). The past experience of coming to understand that experiences while on the ventilator were not real seems to feed into the present experience; the past becomes that against which one tries to make sense of the present, within the very questioning of one’s sense-making abilities. We interpret this as indicating that it is the ability to sense—or discern—what is real and not real that is at stake and is felt as not there or not there as fully as in experiences where the real/not real dimension does not appear as something one cannot trust or take for granted. In this interpretation of this narration, what may be at stake is an existential feeling where “believing,” in the usual sense of taking something “to be the case,” is no longer possible and “the sense of conviction, of what it is for something to be or not be” (Ratcliffe 2011, 64) is temporarily shaken, and this within a temporal horizon where specific past experiences feed into and form the present as a present of uncertainty.

Please note that the above does not imply a causal statement about whether the temporally localized difficulty to sense what is real or not real is an effect of delirium associated with COVID-19 at the ICU, that past studies have noted (for example, studies have pointed to cognitive impairment, anxiety, and depression as parts of post-intensive care syndrome after discharge from ICUs, and that symptoms of cognitive impairment can remain 12 months after discharge) (Pandharipande et al. 2013; Kotfis et al. 2020). The focus of this study is on patients’ experiences, and we understand the experience of difficulty or a breakdown in the ability to sense what is real and not real as a localized and sometimes recurring experience as contributing to making sense of the different experiences of patients who have been hospitalized for COVID-19 and live with symptoms indicative of PCC.

Finally, one more feature is noteworthy: in contrast to the existential feeling of listlessness, the sense of not discerning what is real and not real, as narrated here, had a more localized character; even if it could recur over time, it was not, as narrated here, a temporally *continuous* feeling. While acknowledging this localized character, we suggest that the sense of not discerning what is real and not real still may qualify as an existential feeling since it (as we interpret it) implies an altered relation to the world as a whole within these very experiences, though temporarily discontinuously so. Even so, its more localized character may make this into a subcategory of existential feelings.

## Intentional Affectivity and the Future as Constricted, Fearsome or Lost

Fear was the most commonly described affective phenomenon throughout the interviews, commonly expressed as a fear of not “returning” to one’s previous self or of such a return being painfully slow. In such accounts, fear had a distinct aboutness. To give just one example, Ida underlines that “I want to get back to myself again,” adding that the experience of not improving made her scared: “Something must happen now because, in the end, I might not be able to change this. Do you understand? That is the fear.” Several interviewees also narrate fear as *increasing over time* after being discharged from the hospital, as they experienced that the “return” to one’s previous health status took much longer than they had expected and hoped for. A few interviewees also described how they experienced gradually recovering a few months after being discharged from the hospital and, later, that symptoms again aggravated, and this evoked fear and frustration. This also resonates with past research on how the aftermath of COVID-19 could be experienced as “two steps forward, one step backwards” (Engwall et al. 2022, 4).

Phenomenologically, fear has been described not only as an “unpleasant form of intensity” in the present but also as “project[ing] us from the present into a future” (Ahmed 2004, 65). Building on Heidegger’s analysis of fear as felt in relation to the object that is not yet here but approaches or may approach, Sara Ahmed writes that what we fear “is not simply before us, or in front of us, but impresses upon us in the present, as an anticipated pain in the future” (Ahmed 2004, 65). In our interviewees’ accounts, fear, while unfolding in the present, opens up towards the future in terms of an unpleasantly charged “what if?” (I don’t get better; don’t return to my previous self), which also shapes the present. As one such example, Irene tells of how she does not “dare to take any long strides when walking in the woods. I’m a bit afraid of climbing up on a chair, even though I feel much less dizzy, but the fear actually remains … What if I fall?” Interviewees also narrate fear in relation to the lack of a neatly fixed endpoint to their current situation. As put by Anders when narrating his initial post-admission experience:

I was just shaking when sitting in the shower. I couldn’t hold the shower head because my whole body was shaking, because it requires such a physical effort to sit, and then I became a bit scared. How long will this take?

Ahmed has offered analyses of how fear can be shaped by past experiences, past “histories of association” (Ahmed 2004 63), if or when repeated past experiences of fear gradually feed into and constrain one’s experienced space of possibilities. Another way to express this is to say that fear unfolds in a temporal horizon, and while orienting the self towards that which might happen, it does so through the past as affectively charged and latently present in perception and experience. This helps shed light on interviewees’ narrations of vivid memories of hospitalization as thoroughly and affectively shaping their present. One of the most explicit examples is that of Anna, who describes how passing by the hospital buildings breaks her down emotionally and makes her cry. She says, “I only have to see the intensive care unit, then I feel really crappy, and then this goes away, and then I feel ill and then I howl and I wail and then I feel even worse and experience real unease. I could actually have been dead.”

A few interviewees discuss efforts to manage fear by accepting their slow recovery. This is the case when interviewees describe fear and panic as part of the experience of continuous extreme tiredness and difficulties remembering names or topics in conversations. Again, this can be exemplified by Anders’s narration when he says, “I can’t fool my body and I can’t trick my brain into becoming more active.” This interviewee also narrates acceptance as difficult and describes his fear through a narration that centers on quantifiable time *and* the quantified time as what appears, to him, as breaking his spirit. In his words:

And then it’s the fear that things would remain like this for the rest of my life. It’s almost 15 years until retirement. Every day. There are 365 days a year. 24 hours a day. It’s that experience that I feel, that if one breaks it down like this, then I don’t want to feel like this any longer.

As described earlier, quantifiable time is sometimes being juxtaposed into narrations of what one can no longer do (i.e., into a temporal horizon of felt narrowing possibilities). This is also the case above, with the addition that the quantification is explicitly taken up as lived: it is *this* interviewees’ counted days and hours within a felt constricted space of possibility that is experienced as too long. Further, time is rendered into “explicit temporality,” in Fuchs’s (2005) sense of the term, and in an affective mode through how fear shapes the meaning of the counted days and hours.

While fear is a dominant intentional feeling in the interviewees’ narrations, some of the interviewees also describe how they gradually feel a bit better and hope to have recovered in some months. However, hope, in such narrations, is expressed as a wish for what might be and, thus, exemplifies an affective future orientation towards that which might be possible. While interviewees do not express hope for a full and fast return to their previous self and while some narrate hope of recovery as sometimes lost, they still, from time to time during the interviews, express specific hopes of taking small steps on the route to recovery and gradually becoming stronger. In light of the dominance of fear, this could be interpreted as a gradual and fragile rebuilding or re-formation of distinct hopes, and days with fewer symptoms might allow for such a re-formation. Returning to Ida’s narration of what she can no longer do and how she has lost her joy of living, she also—at one point in the interview—formulates a hope to get “back on the right track.” Hope, in this account, might be understood as a response to seeing what is just outside of one’s radius of possibilities, and on better days, these possibilities may appear as possibly reachable in a not-too-distant future.

## Phenomenological Differentiations within Experiences of Living with Remaining Symptoms after COVID-19 Infection

In this study, we suggest that a phenomenological difference can be discerned in the experience of living with remaining symptoms after COVID-19 through attention to the way intentional and pre-intentional affectivity (specifically existential feelings) help shape the embodied self’s attunement to the world. We understand listlessness and not sensing what is real or not as existential feelings, and they are foundational in the sense that they seem to help shape the felt anchoring, the significance, of the embodied subjects as being-in-the-world. In the accounts of listlessness and not sensing what is real or not, though distinctly different from each other, what is felt, we suggest, is a changed sense of the self-world relation.^9^ Further, while existential feelings as a phenomenological category can be experienced as part of many different situations and experiences of, for example, different illnesses, our point here is that this phenomenological category is helpful when starting to differentiate among features of experiences of living with symptoms indicative of PCC, as these are heterogenous, multifaceted, and not yet well understood. The felt changed sense of the self-world relation, in our interviewees’ accounts, is expressed as related to and part of experiences of living with such remaining symptoms.

However, while more research is needed, the differentiation between phenomenological accounts of experiences of illness where what is experienced is “a whole-sale levelling down of sense and meaning” where “*the degree to which one is attuned to and situated in a world through moods can itself undergo change*” and changes in how one is affected and finding oneself in the world—that is, changes in the “particular mode of attunement in which we find ourselves” (Fernandez 2014, 606 and 598, italics in the original), can help shed further light on some of the results of our study. The feeling of listlessness may be understood as a change in the mode of attunement. If so, it shapes the sense of the self-world relation in such a way that the world and things in it appear as things one cannot bring oneself to care about, but while implying a profound shift in the way of being attuned and finding oneself in the world, this is different from “an erosion or degradation of one’s capacity to be affected,” at all (Fernandez 2014, 607).

Further, interviewees expressed intentional affectivity through various narrations of fear. When this was the case, fear could have thoroughly debilitating effects through how it shaped how situations and the future appear as fearsome and oneself as fearful, and while such fear may permeate many everyday situations (partly depending on what is feared), it was directed to specific things/situations within the world.

Another identified phenomenological differentiation of relevance to understanding experiences of living with symptoms indicative of PCC concerns temporality. The notion of “layered temporality” has been apt to capture the way measurable time of, for example, the daily national updates of numbers of persons being admitted to hospitals for COVID-19 unfolds irrespective of the experience of lived time (compare Howell 2022). Here, however, we use the notion of layered temporalities of the pandemic when referring to the way interviewees quantify time and insert the quantifications, as markers of time that has passed, into narrations of a temporal horizon of narrowing possibilities. In so doing, the quantification seems to underscore the dread of a future that appears as closing down (when experienced in terms of what one cannot and can no longer do) and, at the same time, as too long and too painful to relate to *because* the quantification brings out how what one cannot do is experienced as stretching far into the future.

In addition to layered temporalities, this study highlights the simultaneity of the past, the present, and the future in the lived experience of time. This was exemplified, for example, in the narration of the experience of seeing the hospital after being discharged. In that example, the past was *not* neatly kept in the past, so to say, but explicitly helped shape the present. In phenomenological terms, this temporal simultaneity is commonly expressed as a temporal “thickness:”^10^ the present is not experienced as neatly set apart from the past and future but has, instead, an “immense latent content of the past, the future, and the elsewhere, which it announces and which it conceals” (Merleau-Ponty 1968, 114). Despite the quantifiable time that has passed between being hospitalized and seeing the hospital again, in perceiving the hospital—for one of the interviewees—the “latent content” sprang forth in affectedly strong ways that shaped the experience at the time.^11^ To make use of Merleau-Ponty’s discussion of affectivity and temporality, an “affective texture” can hold sway over experienced things, scenes, and situations and bring out the past as “strangely interwoven” with the present so that the perceived thing—in our case, the hospital—can “condense within itself an entire scene” (Merleau-Ponty [1945] 2013, 53). In the present discussion, this close intertwinement between the past and the present helps explicate the interviewee’s experience of how seeing the hospital where she was hospitalized and close to death brought out the past within the present so that the perceived building could “condense within itself” the experience of being close to death. Of particular relevance to our discussion, this brings out the close intertwinement between affectivity and temporality.

Our analysis brings out how the experience of time is intertwined with affectivity, as in experiences of the future as fearsome or as lost in the sense of the future one hopes for not being experienced as within reach. This intertwinement is also exemplified in terms of how the future no longer has an allure—one cannot make oneself care—in the experience of listlessness and in the feeling of not sensing what is real and not real, where the experienced space and time offers little direction.

However, the analysis in this article exemplifies not only a temporal thickness but also a temporal disruption. As shown in past phenomenological research on severe illness and severe trauma, these can result in a felt “disconnection” of the past, the present, and the future from each other (Papadimitriou and Stone 2011, 2124; Leder 1990). In such experiences, the past no longer helps open up the present and the future as meaningful possibilities: one’s past offers little help in finding one’s way when what one previously enjoyed or hoped for no longer seems viable. Relatedly, the future, in the mode of what is no longer possible, can be experienced as set apart from one’s past field of possibilities: the present can be experienced as a time of dis-orientation, understood as a felt sense of no longer finding one’s way and no longer being able to engage past capabilities and experiences as guidance in the present and towards the future.

Papadimitriou and Stone (2011, 2124) use the term temporal disruption to refer to patients’ experience of no longer being able to “draw readily from their past or their future,” and therefore struggling “to make sense of the work they are being asked to do in rehabilitation and to connect that work to both their past … and their future (how this work will provide a basis for future plans, actions and possibilities)” in the experience of traumatic spinal cord injury (Papadimitriou and Stone 2011, 2124). While theirs is a distinctly different context from that of living with PCC, an acknowledgment of the role of a temporal disruption—the disconnection of past, present, and future from each other—for how to make sense of rehabilitation is also relevant to our context. No longer being able to smoothly draw on the past or the future when making sense or seeking to make sense of the present implies a distinct disruption, apart from the disruption of one’s body-world relation that pain and thorough bodily changes can entail. Even so, however, to make sense of some of the results in this study, the temporal thickness and the temporal disruption need to be held together. This is the case, we suggest, in cases in which the experience of what one “no longer” can do stands forth, in its absence (or in a not-yet mode), as painfully and sharply present, precisely through the way the past of what one could do is vivid in one’s present self-understanding.^12^

In summary, basic phenomenological differentiations within experiences of living with symptoms indicative of PCC can be discerned between pre-intentional affectivity, such as existential feelings, and intentional affectivity, as well as in terms of temporal thickness and temporal disruption. Further, while analytically different, their interrelations are equally important. Finally, in contrast to pandemic scientific narrations of gradual insights and progress despite the uncertainty that characterizes the pandemic, narrations in this study defy not only a neat temporal closure but—for some—also a neat forward direction. A felt loss of direction, a felt dis-orientation when not experiencing or being able to envision a return to oneself, need again to be contextualized against the global and lived uncertainty during the pandemic: while insights and knowledge in the context of COVID-19 gradually emerge, the interviewees continuously live with the insight that little is known, and hope for the future is a hope against the background of uncertainty.

Finally, the study has a few limitations. As stated already in the beginning, no interviewee had been diagnosed with PCC (as the interviews were done before PCC had become an established diagnosis), and we note that more studies are needed to take a stand on the question of whether what we identify in this article is typical or distinct to PCC. Further, the study is a regional Swedish study focused on the early phases of the pandemic. Having said this, we see the results as important to acknowledge as parts of patients’ very heterogenous narrated experiences of falling ill and being hospitalized with COVID-19 and living with remaining symptoms at this early stage of the pandemic—for a better understanding of the heterogeneity of these patients’ experiences. Based on this pilot, we see a need for a thorough analysis of the emergence and establishment of PCC within the Swedish context, as well as further analysis of patients’ experiences of PCC. These are also central foci of the larger project “Biomedicine, Clinical Knowledge, and the Humanities in Collaboration: A Novel Epistemology for Radically Interdisciplinary Health Research and Policy-Work on Post-Covid-19 Syndrome,” which an expanded research team, including the authors of this article, is engaging with.

## Clinical Relevance

This study raises a number of aspects of clinical relevance. Within the context of clinical care, the focus is typically on objective findings, indicating diagnostic pointers and evidence of disease severity. Such findings are sought through physical examination, laboratory tests, and auxiliary methods such as radiology, combined with the medical history obtained in the clinical encounter by interviewing the patient. However, if medical history comes to be restricted to what it is expected to yield in terms of clinically relevant information and if what is sought thereby is based on previous “science and proven experience,” more subtle changes in patients’ self-world relations may be missed. Thus, a large proportion of the scientific literature on COVID-19 has, up to the present, focused on specific symptoms and signs (e.g., dyspnea, muscle weakness, and loss of sense of smell).

As is suggested by the present paper, however, pertinent patient experiences may be of a character such that they may be easy to omit, difficult to inquire, and/or difficult to verbalize, such as pre-intentional, existential feelings and the manner in which distinct intentional feelings can help shape lived temporality. Such feelings were found to be characteristic of patients’ narratives of remaining symptoms after COVID-19 infection, and their analysis contributes added focus, depth, and granularity to the understanding of the patients’ lived experiences, which may be significant in being able to better capture the specific challenges facing persons living with symptoms indicative of PCC and, thereby, in designing and implementing rehabilitation programs apt to assist the recovery of the many survivors of COVID-19 with persisting problems.

Relatedly, and concretely, the study highlights that recovery and adjustment related to symptoms indicative of PCC is not necessarily a linear path but sometimes circular or at least highly fluctuating and, thereby, an affectively challenging processes (as is also the case with some chronic conditions [see Papadimitrious and Stone 2011]), and this insight can again be valuable for clinicians and patients in the recovery and adjustment processes.^13^

## Data Availability

Data supporting this study cannot be made available for research ethical reasons.
